# Cancer and stroke: commonly encountered by clinicians, but little evidence to guide clinical approach

**DOI:** 10.1177/17562864221106362

**Published:** 2022-06-28

**Authors:** Malin Woock, Nicolas Martinez-Majander, David J. Seiffge, Henriette Aurora Selvik, Annika Nordanstig, Petra Redfors, Erik Lindgren, Mayte Sanchez van Kammen, Alexandros Rentzos, Jonathan M. Coutinho, Karen Doyle, Halvor Naess, Jukka Putaala, Katarina Jood, Turgut Tatlisumak

**Affiliations:** Department of Neurology, Sahlgrenska University Hospital, Blå stråket 7, 413 46 Gothenburg, Sweden; Department of Clinical Neuroscience, Institute of Neurosciences and Physiology, Sahlgrenska Academy, University of Gothenburg, Gothenburg, Sweden; Department of Neurology, Helsinki University Hospital, Helsinki, Finland; Department of Neurology, Inselspital, Bern University Hospital, University of Bern, Bern, Switzerland; Department of Clinical Medicine, University of Bergen, Bergen, Norway; Department of Neurology, Haukeland University Hospital, Bergen, Norway; Centre for Age-Related Medicine, Stavanger University Hospital, Stavanger, Norway; Department of Clinical Neuroscience, Institute of Neurosciences and Physiology, Sahlgrenska Academy, University of Gothenburg, Gothenburg, Sweden; Department of Neurology, Sahlgrenska University Hospital, Gothenburg, Sweden; Department of Clinical Neuroscience, Institute of Neurosciences and Physiology, Sahlgrenska Academy, University of Gothenburg, Gothenburg, Sweden; Department of Neurology, Sahlgrenska University Hospital, Gothenburg, Sweden; Department of Clinical Neuroscience, Institute of Neurosciences and Physiology, Sahlgrenska Academy, University of Gothenburg, Gothenburg, Sweden; Department of Neurology, Sahlgrenska University Hospital, Gothenburg, Sweden; Department of Neurology, Amsterdam University Medical Center (UMC), University of Amsterdam, Amsterdam, The Netherlands; Department of Radiology, Sahlgrenska Academy, University of Gothenburg, Sahlgrenska University Hospital, Gothenburg, Sweden; Department of Neurology, Amsterdam University Medical Center (UMC), University of Amsterdam, Amsterdam, The Netherlands; Department of Physiology, Centre for Research in Medical Devices (CÚRAM), National University of Ireland, Galway, Galway, Ireland; Department of Clinical Medicine, University of Bergen, Bergen, Norway; Department of Neurology, Haukeland University Hospital, Bergen, Norway; Centre for Age-Related Medicine, Stavanger University Hospital, Stavanger, Norway; Department of Neurology, Helsinki University Hospital, Helsinki, Finland; Department of Clinical Neuroscience, Institute of Neurosciences and Physiology, Sahlgrenska Academy, University of Gothenburg, Gothenburg, Sweden; Department of Neurology, Sahlgrenska University Hospital, Gothenburg, Sweden; Department of Clinical Neuroscience, Institute of Neurosciences and Physiology, Sahlgrenska Academy, University of Gothenburg, Gothenburg, Sweden; Department of Neurology, Sahlgrenska University Hospital, Gothenburg, Sweden

**Keywords:** cancer, cerebral venous thrombosis, clot, diagnostics, hemorrhagic stroke, intracerebral hemorrhage, ischemic stroke, risk, stroke, therapy

## Abstract

The association between stroke and cancer is well-established. Because of an aging population and longer survival rates, the frequency of synchronous stroke and cancer will become even more common. Different pathophysiologic mechanisms have been proposed how cancer or cancer treatment directly or *via* coagulation disturbances can mediate stroke. Increased serum levels of D-dimer, fibrin degradation products, and CRP are more often seen in stroke with concomitant cancer, and the clot retrieved during thrombectomy has a more fibrin- and platelet-rich constitution compared with that of atherosclerotic etiology. Multiple infarctions are more common in patients with active cancer compared with those without a cancer diagnosis. New MRI techniques may help in detecting typical patterns seen in the presence of a concomitant cancer. In ischemic stroke patients, a newly published cancer probability score can help clinicians in their decision-making when to suspect an underlying malignancy in a stroke patient and to start cancer-screening studies. Treating stroke patients with synchronous cancer can be a delicate matter. Limited evidence suggests that administration of intravenous thrombolysis appears safe in non-axial intracranial and non-metastatic cancer patients. Endovascular thrombectomy is probably rather safe in these patients, but probably futile in most patients placed on palliative care due to their advanced disease. In this topical review, we discuss the epidemiology, pathophysiology, and prognosis of ischemic and hemorrhagic strokes as well as cerebral venous thrombosis and concomitant cancer. We further summarize the current evidence on acute management and secondary preventive therapy.

## Introduction

Cancer and stroke are leading causes of death and disability worldwide. Approximately 40% of all human beings will harbor a malignancy during their lifetimes.^1^ Similarly, approximately 25% will experience a stroke.^2^ Both diseases are devastating with high mortality, morbidity, sufferings, and costs. Fortunately, both cancer and stroke treatments took major leaps during the last two decades. While both diseases represent major global health problems, they may occur in the same individual as several lines of evidence suggest an association between several cancer types and various stroke subtypes. About 10% of patients presenting with stroke have a malignancy.^3^ With aging populations globally, we can expect higher rates already in the forthcoming decades. Patients with malignancies often have similar risk factors as stroke patients, but a clear increase in stroke risks in patients with malignancy occurs without doubt. The mechanisms behind this association are manifold, and not all appropriately explored. From a clinical perspective, an important question is in which stroke patients it is worthwhile and cost-effective to screen for occult cancer. The other relevant clinical question is how to treat patients simultaneously having stroke and cancer, given the increased risk of thrombosis and bleeding at the same time. In the last decades, several encouraging scientific advances in both diagnostics and treatment strategies have greatly changed the course of disease for many patients suffering from cancer or cerebrovascular disease with a prolonged survival rate. This review critically examines the existing data on relationships between cancer and cerebrovascular disease and suggests some approach strategies for clinicians meeting such patients in their practice.

## Association between cancer and different subtypes of stroke

The connection between cancer and stroke is well known.^4,5^ In older studies,^6,7^ ischemic stroke and intracranial hemorrhage were thought to account for equal parts of cerebrovascular disease in cancer patients. A more recent study demonstrated that ischemic stroke is more frequent in cancer patients, however, accounting for approximately 90% of all strokes, similarly to that of the general stroke population.^4^

A nation-wide registry study from the United States confirmed that 1 in 10 hospitalized ischemic stroke patients has co-morbid cancer, and another study showed that about 20% of patients with cryptogenic stroke have occult malignancy at the time of their stroke.^3,8^ Studies restricted to those with active cancer (defined as cancer diagnosis, metastasis of known cancer, recurrent cancer, or receiving cancer treatment, all within 6–12 months before or after stroke onset) report frequencies up to 5% among patients with ischemic stroke. This is significantly higher than the general population.^9–11^ Over the last decades, stroke admissions among patients with cancer have remained stable despite a significant decrease in the general population, and the proportion of patients with concomitant cancer among stroke patients has increased.^3^ This is probably reflecting the positive result of longer life expectancy among the general population, allowing people more time to develop cancer as well as better diagnostics and treatment opportunities for cancer patients improving survival in this group.

In accordance with the study from the United States,^3^ a recently published meta-analysis showed that the pooled cumulative incidence of cancer within 1 year after an ischemic stroke was 13.6 per 1000, being notably higher in studies focusing on cryptogenic stroke and in those reporting cancer screening.^12^ One autopsy study conducted in 1985 indicated that 15% of cancer patients had evidence of cerebrovascular disease upon death.^7^ Several large observational studies have confirmed a substantially increased short-term risk of ischemic and hemorrhagic strokes in patients with newly diagnosed solid or hematological cancers.^4,13–15^ Solid tumors in advanced stage disease of the lung, pancreatic, and colorectal cancers seem to carry the highest stroke risk.^4,11,16^ Other studies reported high incidence of stroke in breast and prostate cancer.^3,9^ (Box 1) A vast increase in stroke risk is also seen in metastatic disease, indicating a more advanced disease.^15^ Stroke can be the initial presentation of cancer^16^ or follow a cancer diagnosis, however,^13^ and the risk of stroke remains elevated even over 10 years following cancer diagnosis.^15^

**Box 1.** The most common types of cancer seen in ischemic stroke, hemorrhagic stroke, and cerebral venous thrombosis.

Ischemic stroke • Lung cancer^a^ • Pancreatic cancer^a^ • Colorectal cancer^a^ • Breast cancer^a^ • Prostate cancer • Gastric cancer • Urinary bladder cancer
Hemorrhagic stroke • Brain metastasis^b^ • Glioblastoma/oligodendroglioma • Leukemia • Lymphoma • Multiple myeloma • Prostate cancer
Cerebral venous thrombosis • Lung cancer • Leukemia • Lymphoma • Colorectal cancer • Breast cancer

a Adenocarcinoma often predominant cancer type.

b From lung, melanoma, breast, and renal cancers.

The clinical features associated with active cancer in patients with acute ischemic stroke (AIS) are the presence of venous thromboembolism (VTE), cryptogenic stroke subtype, and lower frequency of traditional cardiovascular risk factors, for example, diabetes mellitus and high low-density lipoprotein (LDL) cholesterol levels.^9^ Studies investigating TOAST (Trial of Org 10172 in Acute Stroke Treatment) subtypes among patients with ischemic stroke and concomitant cancer reported cryogenic stroke to be the most frequent subtype.^14,17,18^ Furthermore, Cestari *et al.*^19^ reported embolic ischemic stroke to be more common than non-embolic in ischemic stroke patients with underlying cancer, comprising 54% compared with 46%.

The risk of hemorrhagic stroke in patients with cancer has been investigated in a number of studies. A large nation-wide population-based Swedish study showed that cancer patients had 2.2 times increased risk for a hemorrhagic stroke in the first 6 months following a cancer diagnosis.^15^ The risk remained slightly increased (1.2 times) during the following 10 years. Cancers involving the central nervous system, leukemia, endocrine gland, small intestine, and kidney were associated with the highest risk of stroke in this study, and similar results were seen in a more recently published study.^4^

Intracerebral hemorrhage (ICH) is the most frequent type of intracranial hemorrhage associated with cancer.^7,20^ Studies of patients with non-traumatic ICH report a wide incidence range for concomitant cancer, from 0.2% to 15%.^21–25^ The highest incidence was found in a more recently published study from Japan.^21^ Two of the five studies investigating concomitant cancer among patients presenting with non-traumatic ICH excluded patients with previous primary brain tumors and metastatic brain tumors. The incidences of concomitant cancer in these studies were 3.8% and 15%, respectively.^21,24^ Compared with cancer-free patients with ICH, patients with underlying cancer were older, more often male, had received anticoagulation before ICH, had higher prestroke scores according to the Charlson Comorbidity Index, and lower prevalence of diabetes mellitus and arterial hypertension.^24^ Lower hemoglobin (Hb) levels were typically observed in the cancer group, but platelet count was in the normal range similar to the cancer-free study group.^21^

The data from a large US cancer center suggest that 46% of cancer-associated intracranial hemorrhages are caused by coagulopathy and 61% by intratumoral hemorrhage from an intracranial neoplasm.^20^ A single-center cohort study of intracranial neoplasm patients reported a frequency of ICH of 2.4%.^25^ Solid systemic tumors most commonly associated with ICH are lung, melanoma, breast, and renal cancers. This is probably mainly because of their high incidence in the population and frequent metastasis to the brain.^6,20^ Prostate cancer accounted for 5% of ICH in one study.^20^ Among primary brain tumors, glioblastoma multiforme is most often associated with ICH.^6,25^ Among the hematological cancers, leukemia is the one most commonly associated with ICH.^6^ Cerebral venous thrombosis (CVT) is a rare cause of stroke with an incidence of 1.32–1.75 per 100,000 individuals.^26–29^ Not only are malignancies a risk factor of CVT, but also a predictor of poor outcome.^30,31^ The data from cohort studies suggest prevalence of malignancy of 7–10% among patients diagnosed with CVT.^30,32–34^ One case–control study reported increased prevalence of malignancy among CVT patients (53/594, 8.9%) as compared with controls (160/6278, 2.5%) despite younger age among cases.^31^ Cancer types with the highest risk of CVT were lung cancer [adjusted odds ratio (aOR) = 32.4], hematological cancer (aOR = 25.1), gastrointestinal cancer (aOR = 5.8), and breast cancer (aOR = 2.6). The association with CVT was particularly high within the first year after diagnosis of cancer. Another study from the same researchers showed that while cancer history was found in 9.3% of CVT patients younger than 55 years of age, it was 24.4% for those 55 years or older.^35^ Such high probability should alert physicians to remember cancer as a potential underlying factor especially in older CVT patients.

## Potential mechanisms of association between cancer and stroke

As many cancer patients harbor similar demographic characteristics and vascular risk factors as stroke patients, such as older age and smoking, the exact role of cancer underlying a stroke occurrence is not always obvious. Owing to shared risk factors, also cancer patients may have strokes from well-established causes, such as atherosclerosis and small-vessel disease. Furthermore, new-onset atrial fibrillation can be associated with higher rates of occult cancer.^36^ Up to 50% of ischemic strokes in cancer patients can be classified as cryptogenic compared with about 30% in general populations, however, reflecting the uncertainty on their pathogenetic mechanisms.^37^

**Figure 1.**
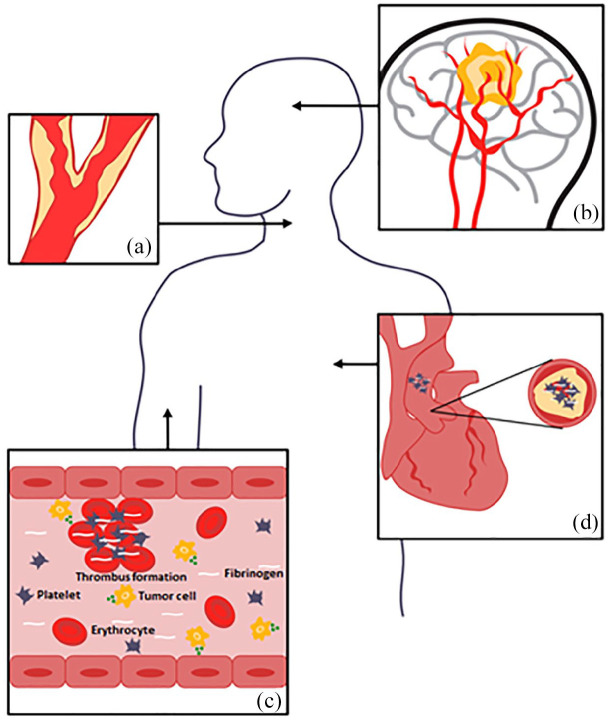
Cancer- and cancer-treatment-related mechanisms that may lead to stroke. (a) Radiotherapy can cause vasculopathy in intra- and extra-cranial vessels by accelerated inflammation and atherosclerosis, especially in patients treated for head and neck cancers. (b) Strokes can occur by mechanical compression of major vessels in the head or neck by invasive tumor growth and spread. Primary brain tumors and brain metastasis can cause intracranial hemorrhage because of intratumoral bleeding, dural vessel rupture because of dural metastasis, or neoplastic venous invasion. (c) Abnormalities in the coagulation system are observed in cancer patients because of cancer itself or as a complication of chemotherapy or surgery. As a result, the risk of both ischemic and hemorrhagic strokes, as well as CVT increases. Tumor cells release tumor procoagulants, such as Factor X, inflammatory cytokines, including tumor necrosis factor alpha, and interleukins 1 and 2 which enhance thrombosis, inflammation, cell proliferation, and vessel vasoconstriction. Disseminated intravascular coagulation characterized by increased levels of D-dimer, prolonged prothrombin time, and low levels of fibrinogen and platelets can lead to both ischemic and hemorrhagic cerebrovascular events. (d) Cancer-mediated hypercoagulability is associated with paradoxical embolism and non-bacterial thrombotic endocarditis, in which sterile valvular vegetations may predispose to distal embolization and stroke. These fibrin–platelet vegetations are almost always located in the left-side heart valves.Furthermore, secondary infections because of immunocompromised can cause endocarditis and mycotic aneurysms increasing the risk of both hemorrhagic and ischemic strokes.

Several cancer-related mechanisms may contribute to the association between an active cancer and stroke. Figure 1 Coagulation system abnormalities are usually considered one of the key players, which increase the risk of both ischemic and hemorrhagic strokes, as well as CVT. Tumor procoagulants – such as Factor X, inflammatory cytokines, including tumor necrosis factor alpha, and interleukins 1 and 2 – enhance thrombosis, inflammation, cell proliferation, and vessel vasoconstriction.^38–41^ Furthermore, cancer cells, especially in pancreas, lung, breast, and colon cancers, release mucins that activate platelets leading to increased thrombosis.^38,41^ The results from histopathologic analysis show that adenocarcinomas are the predominant type of cancer associated with stroke, probably because of its thromboembolic properties.^4,12^ Moreover, especially adenocarcinomas and advanced malignancies may cause disseminated intravascular coagulation that can lead to both ischemic and hemorrhagic cerebrovascular events.^42^ Disseminated intravascular coagulation is usually characterized by increased levels of D-dimer, prolonged prothrombin time, and low levels of fibrinogen and platelets.^42^ Cancer-mediated hypercoagulability is also associated with paradoxical embolism and non-bacterial thrombotic endocarditis, in which sterile valvular vegetations may predispose to distal embolization and stroke.^37,43,44^ These fibrin–platelet vegetations are almost always located in the left heart valves and associated with adenocarcinomas of the lung, pancreas, gastrointestinal tract, or breast.^45,46^ Studies indicate that the disturbance in coagulation and thus the elevated risk of stroke is the highest immediately after cancer diagnosis and then is decreased over time by cancer treatments.^4^

Strokes can occur also by mechanical compression of major vessels in the head or neck, tumor embolism, neoplastic aneurysms from, for example, myxomas and secondary endocarditis, and direct infiltration to bone marrow predisposing to (disseminated) coagulopathy.^47–50^ Both primary brain tumors and brain metastasis can cause intracranial hemorrhage because of intratumoral bleeding, dural vessel rupture because of dural metastasis, or neoplastic venous invasion.^20,25^

In addition to cancer itself, cancer treatments – including chemotherapy, immune checkpoint inhibitors, radiotherapy, and surgery – can increase the risk of subsequent stroke by thrombin generation, radiation-induced vasculopathy, and embolism. Especially platinum-based chemotherapies and inhibitors of the vascular endothelial growth factor signaling pathway are associated with higher risk of ischemic stroke by the release of prothrombotic microparticles from cancer cells and by inducing impaired cell regeneration and further vascular injury.^51–55^ An increased risk of ICH and subdural hematoma (SDH) has been seen in several case series with both children and adults patients with acute lymphoblastic leukemia (ALL) undergoing oral or more often intravenous or intrathecal injection of methotrexate (MTX).^56,57^ L-asparaginase also used for treating ALL is associated with VTE including dural sinus thrombosis and has been linked to hemorrhagic cerebrovascular events.^58,59^ Moreover, invasive cancer surgeries and other procedures like hematopoietic stem-cell transplantation may increase the risk of both ischemic and hemorrhagic strokes.^60–62^

Radiotherapy can cause vasculopathy in intra- and extra-cranial vessels by accelerated inflammation and atherosclerosis, especially in patients treated for head and neck cancers.^38,63,64^ Typically, this vasculopathy develops relatively slowly, leading to an increased risk of artery-to-artery embolic stroke, ischemic stroke because of hypoperfusion or ICH because of vessel disruption due to radiotherapy-induced cavernous angiomas and aneurysms in long-term survivors.^65,66^ Similarly, patients with, for example, lung and breast cancers treated with thoracic radiation are also at a higher risk of developing aortic vasculopathy and subsequent embolic strokes.^49^

Secondary infections are also noteworthy reasons for stroke as most cancer patients are immunocompromised, either because of the primary malignancy or because of cancer treatments.^67^ Infections caused by bacteria, viruses, fungi, molds, and parasites can cause systemic infections, brain abscesses, endocarditis, mycotic aneurysms, and vasculitis.^60^ All of these may be associated with both ischemic and hemorrhagic strokes. Finally, the association between cancer and stroke might also be because of increased psychological stress heightening the risk of stroke.^68^ In some high-risk stroke patients, concerns for bleeding may also lead to discontinuation of antithrombotic treatments increasing the risk of ischemic cerebrovascular complications.

It has been suggested that the risk of stroke in patients with cancer resembles a *U*-shaped curve in which the risk of stroke recurrence is the highest soon after cancer diagnosis when the cancer is very active and associated with a high risk of coagulation disturbances, together with the above-mentioned additional risks when cancer treatment is being introduced. The risk is then reduced for several years if the cancer is being controlled and then followed again by a rise in stroke risk because of long-term effects of cancer treatments mostly because of vasculopathies from radiation or possibly cancer relapse.^49^

## Clinical, laboratory, and imaging patterns: how cancer-associated strokes differ from other strokes?

### Clinical characteristics

Although many patients with cancer and stroke have advanced disease with known metastases, patients with early-stage cancers can also develop stroke.^4,37^ In addition, AIS can even be the initial manifestation of cancer.^50,69,70^ General symptoms and signs that could point toward occult cancer are unintentional weight loss, fatigue, unusual bleeding, newly appeared lumps and nodules, persistent cough, blood in sputum, subfebrile body temperature, or fever. The presence of these symptoms and signs should alert physicians about possibility of cancer in any patient. (Box 2)

**Box 2.** Signs and symptoms that could point toward concomitant cancer in patients with AIS.

Clinical • Age greater than 65 years • Weight loss • Smoking history • Subfebrility/fever • Occurrence of venous thromboembolism • Blood in sputum • Lack of traditional AIS risk factors (diabetes, hypercholesterolemia) • Cryptogenic stroke subtype
Laboratory • D-dimer ↑ • Fibrinogen ↑ • CRP ↑ • Hemoglobin ↓ • Albumin ↓
Imaging • Multiple territory infarctions on MRI-DWI sequence • Absence of dense vessel sign because of different clot histology

AIS, acute ischemic stroke; CRP, C-reactive protein; DWI, diffusion-weighted imaging; MRI, magnetic resonance imaging.

Some studies report slightly younger age among patients with AIS and active cancer compared with AIS patients without cancer diagnosis,^10^ although the opposite was observed in other studies.^9,14^ In addition, they have more severe strokes [higher National Institutes of Health Stroke Scale (NIHSS) score] at admission^10,71^ and more often multiple infarctions than AIS patients without a cancer diagnosis (26–50% *versus* 5.2–10%).^10,71^ AIS patients with cancer are more prone to develop early neurological deterioration and suffer in-hospital death than patients without a cancer diagnosis.^4,10^ In one study, in-hospital death was as high as 21.9% among patient with active cancer and ischemic stroke.^10^ A higher incidence of carotid stenosis has been found in patients treated with radiation therapy targeting the head and neck.^72^

### Laboratory characteristics

Several studies have reported elevated D-dimers and fibrin degradation products in patients with AIS and active cancer.^11,14,73–76^ Gou *et al.*^75^ tested whether D-dimer levels could predict cancer in patients with AIS. The normal range in that study was D-dimer less than 0.55 mg/l. Approximately two-thirds of the patients with active cancer had D-dimer greater than 1.55 mg/l, compared with only 8% of the patients without active cancer. When D-dimer of greater than or equal to 0.55 mg/l was used as the cutoff level, the test had fair sensitivity (80%) but inadequate specificity for cancer-related stroke (67%). When they included both D-dimer of greater than or equal to 0.55 mg/l and multiple territory infarctions on imaging studies, the specificity for cancer-related stroke raised to 99.7%. The sensitivity was low, at only 24.5%, however. When D-dimer of greater than or equal to 5.5 mg/l was used as the cutoff value, the test yielded a high specificity (99.6%) regardless of multiple territory infarctions were present or not. The authors concluded that extraordinarily high D-dimer or combination of high D-dimer and multiple territory infarctions may be used to detect malignancy in patients with AIS. Except for elevated D-dimer and fibrin degradation products, patients with active cancer may have higher levels of C-reactive protein (CRP) and lower levels of cholesterol and Hb.^11,76–78^ Lee *et al.*^76^ demonstrated that stroke patients without active cancer had a median CRP concentration of 5 mg/l at admission compared with 9 mg/l in patients with active cancer (*p* < 0.001). In agreement with that study, Cocho *et al.*^77^ showed similar results with median CRP concentrations of 18 mg/l in patients with occult malignancy presenting with a stroke *versus* 5 mg/l in stroke patients without occult cancer (*p* = 0.001). They also reported higher fibrinogen levels in patients with occult cancer. The authors suggested that patients with an AIS and CRP greater than 20 mg/l or fibrinogen greater than 600 mg/dl should be examined for occult malignancy, especially in patients with undetermined stroke etiology.

### Imaging characteristics

Studies suggest that specific findings with [(micro-)embolic scattering of diffusion-weighted imaging (DWI) lesions in multiple vascular supply territories and in variable stages] on DWI in magnetic resonance imaging (MRI) can indicate underlying cancer in some stroke patients, as multiple acute cerebral infarctions on imaging studies are more common in patients with cancer-associated stroke.^79^ In one retrospective study, comparing ischemic stroke patients with underlying malignancy to ischemic stroke patients with atrial fibrillations as etiology, bilateral anterior circulation, and posterior circulation infarcts on MRI-DWI sequences, so-called ‘Three territory sign’, was highly suggestive of underlying malignancy.^80^

The composition of clot in cancer patients has been shown to differ having a higher platelet/fibrin fraction compared with clots in cancer-free patients^81,82^ which could change the attenuation of the clot in the computed tomography (CT) scan leading to the absence of dense vessel sign.

In ICH, abnormal mass effect/peri-hemorrhagic edema not explained by the intraparenchymal blood clot and multi-focal bleeding sites seen on brain CT scan may point to underlying brain tumor and metastasis.^6^ In these cases, brain MRI may reveal an underlying, structural solid brain tumor. This is a field in which evidence-based data are scarce, however. Likewise, the data on specific neuroimaging characteristics indicating underlying cancer in patients presenting with CVT are lacking.

## Prognosis in patients having both cancer and stroke

### Mortality

Prognosis is usually poor among people with concomitant active cancer and AIS, with increased stroke severity, early neurological deterioration, and up to tripled risk of death during hospitalization.^9,10,49^ Stroke severity, active cancer, metastases, cryptogenic mechanisms, diabetes, elevated D-dimer, and CRP are predictors of mortality in cancer-associated stroke.^21,49,83^ The cumulative risk of death over 16-year follow-up in one study was more than three times higher among patients with a cancer diagnosis compared with cancer-free stroke controls.^83^ The cancer types most strongly associated with death were melanoma and lung or other respiratory tract cancer. Some studies have reported favorable short-term outcomes in AIS patients with concomitant cancer, but long-term functional outcome and survival are reduced.^37^ Moreover, short- and long-term survival depends on the cancer type, that is, whether the cancer is defined as active and whether metastasis is present.^84^

The prognosis of ICH in cancer patients is generally poor with 30-day mortality up to 31% and a median survival of 3 months.^20^ Whether the prognosis for ICH in cancer patients is worse than in the non-cancer ICH patient population is still a matter of debate, however. Previous studies have shown no difference in ICH patients with and without cancer with respect to in-hospital mortality, short- and long-term outcome, and the proportion of patients with favorable functional outcome over a period of 12 months after ICH.^20,85^ A possible reason why additional cancer diagnosis did not impact long-term prognosis after ICH was likely the importance of the severity of the bleeding itself.^85^ Other studies have shown, on the contrary, higher odds of death and lower odds of favorable discharge in ICH patients with cancer.^21,24^ Patients with hematopoietic tumors have the highest rate of death during hospitalization, and in line with this, coagulopathy as etiology of ICH showed the shortest survival rate.^20,24^ A high proportion of coagulopathy has been reported in patients with hematologic tumors.^21,24^ In addition, patients with metastatic tumors had worse outcome with twofold increased odds for death as compared with ICH patients without cancer in one study.^24^ Impaired consciousness, not having a primary brain tumor, multiple foci of hemorrhage, hydrocephalus, pharmacological treatment for increased intracranial pressure (ICP), and not receiving ventriculostomy were significant predictors of 30-day mortality among patients presenting with ICH and cancer.^20^ Similar to findings in AIS and ICH, studies have shown that concurrent cancer and CVT were associated with worse outcome with higher modified Rankin scores and a threefold risk for death and dependency compared with CVT with no concurrent cancer.^30,86,87^

### Functional outcome of stroke in survivors

Short-term outcome can be favorable in cancer patients with stroke according to a few studies, reporting 44% of patients with a modified Rankin Scale (mRS) 0–2 at discharge.^78^ In a cohort study consisting of 263 patients with active cancer and stroke, the median mRS score at hospital discharge was 3 (interquartile range = 2–5).^37^ On the contrary, another study reported significantly increased odds ratio for functional dependency in patients with underlying cancer at discharge and 3-month follow-up compared with cancer-free patients.^88^

### Recurrent rates

Patients with active cancer are more likely to experience a recurrent stroke than stroke patients without a cancer diagnosis.^10,88,89^ In a retrospective case–control study,^89^ stroke recurrence within 1 year after the index stroke was noted in 28% of the patients with cancer, compared with 13% of control patients free of cancer. Childhood cancer survivors have a high long-term rate of stroke and stroke recurrence.^90^ In one study, the 10-year cumulative incidence was 21%, and in patients treated with cranial radiation over 50 gray, the 10-year cumulative incidence was 33%.^91^ Another study conducted at a cancer center investigated the rate of recurrent thromboembolic events after AIS in patients with active cancer. In this cohort, 31% of the patients were diagnosed with a recurrent thromboembolic event by 3 months, including 13% with recurrent AIS, which is nearly threefold higher than the recurrent stroke rates in the general stroke population.^37^ Adenocarcinoma cancer histology predicted for recurrent thromboembolism.^37^ The increased recurrence rate might not be universal as one study with younger cancer patients aged 15–49 years could not confirm an increased recurrence rate, however.^83^

## Which stroke patients are good candidates for screening for cancer?

### Ischemic stroke

As not all stroke patients can be extensively investigated for presence of an underlying cancer, many studies looked at the factors that suggest the presence of cancer underlying the index ischemic stroke. In these studies, male sex, older age, and a history of smoking were more often present in patients with occult cancer suffering a stroke than in the stroke population without cancer.^14,16^ Regarding stroke features typically associated with occult cancer, undetermined stroke etiology and involvement of multiple vascular territories on neuroimaging are the most important.^12,18^ Markers of inflammation such as increased levels of CRP, hypoalbuminemia and anemia, and markers of upregulated coagulation activity, mainly elevated D-dimer, fibrinogen monomer, and fibrinogen were associated with cancer after stroke.^11,12,14^ International guidelines do not provide an answer to the question of patient selection for cancer investigations, but one probability score developed for ischemic stroke patients may help in clinical practice.^11^

For patients younger than 75 years, a score assigning one point for each of increased D-dimer, lower Hb, and a history of smoking has been constructed.^9^ The score total is 3 points as follows:

- D-dimer greater than or equal to 3 mg/l (1 point).
- Hb less than or equal to 12.0 g/dl (1 point).
- History of smoking (1 point if yes).

Assuming cancer prevalence to be 5%, calculation showed that the probability of active cancer was 13% in patients who scored 2 points and 53% in patients who scored 3 points.^11^ For a patient with 2 or more points, cancer screening is therefore warranted if no cancer is diagnosed prior to stroke. Many centers, but not all, measure D-dimer routinely on admission in stroke patients. With the increase in cancer and increasing life expectancy in general and given that D-dimer is an inexpensive blood test, it may be reasonable to include D-dimer measurement to routine testing in stroke patients on hospital admission. Idiopathic D-dimer increases with age, and thus becomes a less specific marker of coagulation in older patients.^92^ Nevertheless, D-dimer is a direct marker of activated coagulation and has therefore been suggested by some to be used alone for cancer screening of stroke patients.^93^ The breadth of cancer investigations depends on local resources and may include less sensitive and less specific approaches such as detailed history-taking and clinical examinations associated with simple laboratory tests and X-ray films or enriched toward more sensitive and specific tests such as whole-body CT imaging and tumor markers.

## Clot analysis

Two novel lines of research may help developing technologies that serve as cancer diagnostic screening tests in stroke patients. Clots retrieved during thrombectomy in AIS patients with large-artery occlusions can be analyzed for macroscopic and microscopic properties. First, cancer-related ischemic strokes are predominantly embolic,^19^ and it is known that cardioembolic and cryptogenic clots are more fibrin- and platelet-rich than those of atherosclerotic etiology.^94,95^ Genomic and proteomic analyses of clot retrieved by thrombectomy offer a useful window to investigate cancer-related hypercoagulation. Second, blood contains many circulating proteins and nucleic acids, including cell-free DNA, which in cancer patients can include circulating tumor DNA. Screening technology based on the tumor-specific mutations and methylation patterns in circulating tumor DNA could allow early diagnosis of a broad range of cancers using a single blood test.^96^ Such liquid biopsy screening has the capacity to identify cancer type and stage with very high selectivity and sensitivity, particularly in combination with a panel of protein biomarkers.^96^ How precise and cost-effective these approaches will become in the future is not yet clear, however.

## ICH

While intratumoral bleeding is a potential complication of any brain tumor, according to a registry-based study with 208 patients with hemorrhagic stroke and cancer, both ICH and subarachnoidal hemorrhage were most often associated with extracranial solid tumors (68% of all included patients), while only 16% had primary brain tumors and 16% hematopoietic tumors.^20^ Most common solid tumors associated with intracranial bleeding were breast, melanoma, lung, and renal carcinomas.^6^

Cancer as a potential cause in patients with ICH should be suspected in the following scenarios: (1) absence of classical risk factors for ICH (e.g. arterial hypertension), (2) history of symptoms with inexplicable weight loss, or (3) abnormal mass effect/peri-hemorrhagic edema not explained by the intraparenchymal blood clot and multi-focal bleeding site (e.g. multi-focal metastasis). In these patients, brain MRI is necessary for further investigation. If coagulopathy from non-brain tumor is suspected, appropriate investigations are required.

## CVT

Given the association between malignancies and CVT, screening of CVT patients for malignancies may be indicated with the rationale of diagnosing the cancer at an early and potentially treatable stage especially when considering the fairly young age of most CVT patients. Furthermore, active cancer could justify longer treatment with anticoagulation. No study has yet investigated how general or targeted screening would affect outcome, however. Consequently, given the very low evidence supporting any decision on this matter, the current international guidelines do not recommend routine screening for occult malignancy in patients with CVT to improve outcome.^97,98^ In selected CVT patients with high risk of malignancy, however, non-invasive low risk and cost-effective screening methods could guide the clinician to selecting patients for more thorough investigations. Although not specifically studied in CVT patients, general risk factors for cancer most likely apply to patients diagnosed with CVT such as higher age, smoking, estrogen use, heredity, and sun exposure. Furthermore, male sex and absence of headache have been associated with higher risk of cancer among patients with CVT.^99^ Studies of screening of occult malignancies in unprovoked VTE at other locations are in favor of limited screening strategies.^100,101^ Thus, despite the lack of sufficient evidence, it seems reasonable to also perform a limited screening for cancer in patients diagnosed with CVT of unknown etiology. Physicians should be more prone to investigate CVT patients without general risk factors or potential symptoms of cancer, especially with increasing age.

## Acute phase treatment in patients with stroke and cancer

Efficacy and safety data for intravenous thrombolysis (IVT) in patients with active cancer are scarce. Available evidence on this issue is mainly limited to case series and non-randomized cohort studies that are probably also further hampered by publication bias. The American Heart Association and American Stroke Association guidelines for the management of AIS^102^ state that the safety of IVT in patients with concurrent malignancy is poorly established, but the guidelines still provide instructions based on expert opinion consensus. This guideline states that IVT is contraindicated in both gastrointestinal cancer and in intra-axial intracranial tumors, but not contraindicated for patients with extra-axial intracranial tumors or other systemic malignancy with at least 6 months of remaining life expectancy.

A meta-analysis published in 2020^103^ showed comparable IVT outcomes between AIS patients with active cancer and AIS patients without a cancer diagnosis in terms of proportion with (1) favorable neurological outcome, (2) symptomatic ICH, (3) major bleeding, and (4) 3-month mortality. Furthermore, no difference was observed between patients with gastrointestinal malignancy and those with other malignancies. By contrast, another meta-analysis from 2021^104^ raised safety concerns about symptomatic ICH rates in patients with active cancer who received IVT. In this latter study, the symptomatic ICH risk in patients treated with IVT was 10-fold higher among active cancer patients. Differences in study selection criteria between these two contemporary meta-analyses may explain the discrepancy. The first meta-analysis^103^ included case series, whereas the second one^104^ excluded them.

In a study based on a large data set including 32,576 AIS patients treated with IVT, 807 also had active cancer.^105^ Patients with hematologic malignancies, solid tumors without metastasis, and metastatic cancer were included. IVT in patients with cancer was neither associated with increased risk of ICH nor in-hospital mortality. Subgroup analysis on different cancer subtypes showed that solid and metastatic tumors were associated with lower home discharge rates and higher in-hospital mortality than hematologic malignancies while the cancer subtype had no impact on symptomatic ICH rates.

In a large retrospective cohort study with over 40,000 AIS patients treated with IVT, 93 patients had a gastrointestinal malignancy. In this study, no symptomatic ICH rate difference was observed (4.5% in patients with gastrointestinal cancer *versus* 5.1% in patients without cancer). Moreover, IVT-related serious complications were overall comparable between gastrointestinal tract cancer patients and patients without cancer (9.0% *versus* 9.4%).^106^

In patients with intracranial tumors, it is important to distinguish whether the tumor localization is intra- or extra-axial. A review paper from 2021^107^ reported results from 25 cases involving patients with intracranial extra-axial tumors (predominantly meningioma) and concomitant AIS who received IVT. Among these, no complications were reported, suggesting that IVT could be safely offered to such patients. The outcomes for five patients with intra-axial tumor treated with IVT were also reported in the same review. One of these patients (with glioblastoma) suffered a symptomatic ICH following IVT treatment. Reports on IVT safety within the context of cerebral metastasis are completely lacking.^107^ Thus, there is not enough evidence to support the use of IVT in patients with AIS and simultaneous intracranial intra-axial tumor or metastases.

Endovascular treatment (EVT) in patients with AIS because of large vessel occlusion up to 24 h from stroke onset is well-established since the publication of randomized controlled studies in 2015 and 2018.^108,109^ Patients with a known cancer were excluded from the randomized studies, and the only available evidence for EVT of large vessel occlusion in cancer patients is coming from case series and retrospective cohort analyses. While in two of three series, the recanalization rate is similar to the rate in cancer-free patients,^110,111^ the clot in the cancer patients might be more difficult to retrieve.^112^ A recent study utilizing the MR CLEAN Registry reported that 124 (4.8%) of 2583 patients who underwent EVT because of AIS had active cancer. The active cancer group had worse prestroke disability level with even one-fourth already being assigned to palliative care.^113^ Despite similar successful recanalization and symptomatic ICH rates, the active cancer group had significantly higher disability and mortality as well as higher recurrent stroke rates at 90 days.^113^ When active cancer patients on palliative care were analyzed separately, 90-day mortality in this group reached over 80%.^113^

The clots in cancer patients have been shown to have higher fibrin/platelet composition^81,82^ which is associated with increased procedural time^114^ and poorer recanalization rate.^115^ Moreover, cancer patients might present with occlusions in multiple vessels in different vessel territories^71^ which can lead to inadequate recanalization because of unreachable distal clots or to futile recanalization because of already impaired collateral supply secondary to multiple occlusions. Another characteristic of the cancer patients with a large vessel occlusion is the occurrence of tumor emboli as cause of intracranial occlusion instead of a pure blood clot. While anecdotal cases of such emboli have been published in which such a clot was extracted,^116^ these clots may be difficult to extract in the first pass and a combination of different techniques or multiple passes might be needed for a successful recanalization.^117,118^ However, the frequency of tumor embolism among all cancer-related large vessel occlusion AIS patients is unknown and is probably small.

A striking difference has been shown in the late neurological outcome at 3 months with a significantly worse outcome in the cancer patients treated with EVT but also in the occurrence of any postoperative bleeding.^110,111,119^ This was evident even in patients where the early neurological outcome (NIHSS at 24 h or mRS at discharge) was similar with the cancer-free patients treated endovascularly^110,112,120^ or patients with favorable recanalization and low infarct volume postoperatively.^121^

While most of the deaths were shown to be cancer-related and not associated with the AIS, one could argue that EVT can be safe and effective in these patients. Caution should be taken, however, in selecting candidates for EVT of large vessel occlusion in the presence of cancer as this is a very heterogeneous group in which patients with relatively good status co-exist with patients who have major co-morbidities and short life expectancy. In daily practice, in most comprehensive stroke centers, patients presenting with a large vessel occlusion and significant neurological deficit are usually not excluded from an EVT if they meet all other criteria. Patients on palliative care probably represent a subgroup, however, in which EVT may not improve outcomes, although even there still exceptions might exist because of heterogeneity. A more tailored and individualized approach in selecting such patients should be encouraged taking in account the differences in late outcome (3 and 12 months).

## Long-time secondary preventive treatments in patients with stroke and cancer

In addition to treatment of cancer itself, secondary stroke prevention in cancer patients should primarily be directed toward the specific ischemic stroke etiology and focusing on the management of modifiable risk factors. As many ischemic strokes in active cancer patients may be labeled as cryptogenic, however, decisions on optimal secondary prevention in practice are more complex. Cryptogenic strokes in patients with cancer usually have an embolic pattern on brain imaging, leading to speculations that they arise from proximal sources such as from the venous system through paradoxical embolism or from left atrium or left atrial appendage in cancer-mediated hypercoagulopathy.^49^

Although several papers suggest that low-molecular-weight heparin (LMWH) may be suitable for cancer and VTE,^122^ only a few studies, thus far, have conducted head-to-head comparison between different anticoagulation regiments (e.g. parenteral *versus* oral) or between anticoagulation and antiplatelet treatment in patients with cancer and stroke.^123–125^ Therefore, the optimal antithrombotic therapy for these patients is still unclear, and no international guideline has included a recommendation on this topic yet.^18,126^

In one study, lower fibrinogen and higher D-dimer levels were associated with higher frequency of embolic signals detected by transcranial Doppler ultrasound in patients with cancer and AIS, and the initiation of anticoagulation treatment lowered D-dimer levels significantly.^127^ Furthermore, another retrospective single-center study including patients with cancer-associated strokes suggested that enoxaparin would be more effective in lowering D-dimer levels compared with warfarin with no differences in the rates of major bleeding.^128^ However, both these studies were limited by a small sample size and the level of D-dimer served merely as a surrogate marker for clinically relevant endpoints such as recurrent ischemic stroke. Furthermore, a recent observational study in 48 patients with cryptogenic ischemic stroke and active cancer compared LMWH with direct oral anticoagulants (DOACs) and showed similar clinical outcomes and safety profile between the treatment arms.^124^

Investigating the superiority of anticoagulation to antiplatelet treatment, the Trial of Enoxaparin *versus* Aspirin in Patients With Cancer and Stroke (TEACH) pilot trial randomized 20 cancer patients with a recent ischemic stroke to enoxaparin or aspirin arm. The study showed no differences in the cumulative rates of recurrent thromboembolic events, major bleedings, or survival between the groups.^125^ In an exploratory analysis of the New Approach riVaroxaban Inhibition of Factor Xa in a Global trial *versus* ASA to prevenT Embolism in Embolic Stroke of Undetermined Source (NAVIGATE ESUS) trial, a slightly lowered dose of rivaroxaban (15 mg once daily) was compared with aspirin (100 mg once daily) in patients with a history of cancer and a recent embolic stroke of undetermined source. The trial did not show a benefit of rivaroxaban over aspirin, but, in contrast, there were more major bleedings in the rivaroxaban group. The trial itself was not, however, designed for this purpose.^129^ Indeed, potential benefit of any antithrombotic treatment might be outweighed by the increased risk of bleeding complications in this patient population and larger prospective randomized trials comparing various anticoagulant and antiplatelet agents are needed.

Patients with cancer are at increased risk of thrombosis, and there have been concerns that oral anticoagulation may increase the risk of ICH in these patients, especially in those with solid brain cancer. Recent reports and reviews^130^ refined our understanding of the bleeding risk associated with different types of brain tumors with melanoma and renal cell carcinoma-metastasis being at the highest risk for ICH. In major trials investigating DOACs, there were only a small number of patients with malignancies and subanalyses did not find any safety concerns.^130^

Management of patients with cancer and ICH is challenging and mostly related to the presumed cause (e.g. coagulopathy *versus* intratumoral hemorrhage). Due to lack of studies, we can only recommend antihypertensive treatment to keep blood pressure under 160 mmHg in the first days as in line with standard treatment for ICH.

## Anticoagulant treatment of cancer-associated CVT

Current guidelines provide no specific recommendation on the anticoagulant treatment of patients with cancer-associated CVT.^98,131,132^ Patients with cancer-associated CVT were neither included in the randomized trials on anticoagulant treatment of CVT^133,134^ nor in the trials on anticoagulant treatment of patients with cancer-associated VTE.^135^ As observational data on this topic solely come from case reports and case series,^136^ for the most part, we must extrapolate data from other cancer-associated VTE.

LMWHs have long been the treatment of choice for patients with cancer-associated VTE.^135^ Recently, several randomized trials have compared DOACs with LMWH for the treatment of cancer-associated VTE. DOACs were associated with a similar risk of recurrent VTE, and a similar^137,138^ or higher^139^ risk of major bleeding compared with LMWH. A higher risk of major bleeding was seen only among patients with gastrointestinal or urogenital cancer. Following these results, many guidelines now suggest the use of DOACs (apixaban, edoxaban, or rivaroxaban) over LMWH for cancer-associated VTE in patients who do not have gastrointestinal or urogenital cancer, contraindications for DOACs, or drugs that significantly interact with DOACs.^132,140^ LMWH remains the anticoagulant of choice for patients who meet one of these criteria. Even though extrapolating evidence from cancer-associated VTEs may be considered, nevertheless CVT represents a unique disease entity, not in the least because approximately 40% of patients present with hemorrhagic intracranial lesions.^30^ Studies on the best treatment approach for this specific patient group are thus still much needed.

## Conclusion

Over the last years, great steps have been taken both in our knowledge of treating cancer and stroke, and of our understanding of the underlying mechanisms in how cancer can cause stroke. At present, additional evidence is still warranted to more accurately identify specific subgroups of cancer patients and distinguish those having higher risks to suffer a stroke from those with low risk. By improved classification of patients as high or low risk and thereto understanding the potential different mechanisms underlying the stroke event in the specific patient, the decisions that are more correct on how to best optimize secondary antithrombotic preventive therapy with either antiplatelet therapy or anticoagulants could be made. Further studies are also needed to evaluate the acute therapy in stroke complicating cancer considering the potential risks but also the great benefits with thrombolysis and thrombectomy, as well as studies of risks and benefits of cancer screening in patients with acute stroke and CVT.

In clinical practice, conventional etiologies and risk factors should be evaluated in a stroke patient with known or newly diagnosed cancer as cancer patients may harbor one or more of the common causes for stroke and traditional vascular risk factors. These additional examinations should be undertaken if the patient is deemed to have prognostics enough to motivate active care. Adding D-dimer measurement to the routine laboratory package of all stroke patients may be considered in order to find occult cancer among patients with acute stroke.
